# Identification of Panoramic Photographic Image Composition Using Fuzzy Rules †

**DOI:** 10.3390/s24041195

**Published:** 2024-02-12

**Authors:** Tsorng-Lin Chia, Yin-De Shin, Ping-Sheng Huang

**Affiliations:** 1 Department of Applied Artificial Intelligence, Ming Chuan University, Taoyuan City 333, Taiwan; tlchia@mail.mcu.edu.tw (T.-L.C.); s7160975@gmail.com (Y.-D.S.); 2 Department of Electronic Engineering, Ming Chuan University, Taoyuan City 333, Taiwan

**Keywords:** panoramic image, feature extraction, photographic composition, fuzzy rules

## Abstract

Making panoramic images has gradually become an essential function inside personal intelligent devices because panoramic images can provide broader and richer content than typical images. However, the techniques to classify the types of panoramic images are still deficient. This paper presents novel approaches for classifying the photographic composition of panoramic images into five types using fuzzy rules. A test database with 168 panoramic images was collected from the Internet. After analyzing the panoramic image database, the proposed feature model defined a set of photographic compositions. Then, the panoramic image was identified by using the proposed feature vector. An algorithm based on fuzzy rules is also proposed to match the identification results with that of human experts. The experimental results show that the proposed methods have demonstrated performance with high accuracy and this can be used for related applications in the future.

## 1. Introduction

Due to the rapid development of electronic technology, using personal devices such as smartphones and digital cameras to take photos and videos is becoming increasingly popular. Also, guiding the user to make panoramic images has been included as an essential function inside those personal devices because the panoramic image can provide wider and richer content than regular images. The panoramic image made from multiple photos or video frames becomes another feasible solution for the increasing demand to watch the complete scene from a single photo.

The feasible approaches to generate an esthetically acceptable panoramic image include the following solutions: (1) The camera gives guidelines on the control screen to direct the user to modify his viewing angle and rotate the camera during the shooting stage; (2) The panorama editing software provides recommendations for the user during the editing stage; (3) The quality evaluation system makes the esthetic judgment automatically for generating a panoramic image with high esthetic value. It is, therefore, essential to learn how to define a panoramic image with an esthetic and pleasing picture. Most professional photographers indicate that photographic composition is critical to making excellent esthetic photos. However, the techniques to evaluate and generate a panoramic image with high esthetic quality are still deficient.

Apart from the approach selection for making panoramic images, another issue is judging the esthetic value of a panoramic image. For normal photographers, it is hard to ensure that the panoramic image generated has esthetic value or that any decision mechanism of esthetics exists. For an image that is comfortable for human eyes, the photographer must let the viewer know which subject is emphasized and make the whole picture with extension and balance. Also, a certain size ratio must be satisfied between the main subject and the background scene. The critical factor for those issues is decided by the photographic composition of this panoramic image. However, only the principle of photographic composition for traditional photos is currently studied; the corresponding principle for panoramic images is still deficient. Therefore, the panoramic images and their photographic composition are analyzed in this paper. The composition modes are summarized and used to identify the mode of photographic composition for panoramic images.

According to the evaluation results of photographic composition for traditional photos, this paper adopts the features of color, shape, and geometry extracted from each panoramic image to classify the mode of photographic composition into one of five types. In addition, because professional photographers suggest that a good picture may have more than one type of photographic composition, the identification algorithm based on fuzzy rules is also proposed to match the identification results with that of human experts.

This paper is organized as follows: Section 2 introduces photographic composition types and photographic composition analysis. The proposed algorithms are elaborated in detail in Section 3. The experimental results regarding how to generate panoramic images and composition identification are shown and explained in Section 4. Some conclusions are described in Section 5.

## 2. Related Works

To generate a panoramic image from multiple photos or video frames, the motion vectors between adjacent images must be calculated first. The movement of image pixels caused by camera and object movement can be divided into local and global motion. The local motion information is mainly extracted from the results of object segmentation. For example, the object’s displacement calculated from the segmentation results is used as the local motion information [1,2]. The hierarchical model of motion estimation [3] is adopted by Zhu et al. [4] to analyze the motion inside the image. The processing steps include pyramid creation, motion estimation, image warping, and coarse-to-fine analysis.

The aim of extracting global motion information is to locate the position of each image inside the panoramic image. Rav-Acha et al. [5] adopt the dynamic texture and the moving object in the image to calculate the image pixel movement caused by camera motion. Furthermore, min-cut optimization is proposed by Agarwala et al. [6] to select video fragments and image stitching is used to create the panoramic image both spatially and temporally. However, this method cannot handle the case of moving objects inside the scene. To tackle this problem, Chen et al. [7] present the technique of combining mosaic-based temporal and color-based spatial segmentation. The color of the background is decided as the most frequent color appears at the same position. Then, the moving object can be removed by distinguishing the background color from the object color. Also, Burt and Adelson [8] proposed using a multiresolution spline to blend multiple color channels to eliminate the visible edge between the border of mosaicking two images. Nevertheless, during the stage of shooting, in the panoramic image, due to the change in position and the brightness of the light source, both the color and the brightness on the left and the right sides are inconsistent. Also, uneven exposure and halo problems will occur.

The purpose of photographic composition is to demonstrate the visual balance of the whole photo and attract the viewer’s attention to the main subject by arranging proper locations for subjects in the photo. For traditional images, many types of photographic composition rules have been generalized [9]. Also, valuable guidelines are provided by some photographers to assist the users in making photos more exciting and engaging. Hence, understanding the principle of photographic composition is required for a photographer. By designing the camera function to guide the photographer in taking a decent photo, the identification method for different types of photographic compositions should be included. However, the automatic identification for photographic composition still focuses on the center or sun-like composition [10,11], which arranges the main subject in the center of the image with a misty background.

Furthermore, the rule of thirds or golden mean composition is studied by placing the main subject in specific photo positions to draw visual attention [12,13]. The photographic composition also investigates the skyline’s role and the horizon [14]. Although the rules of photographic composition for traditional photos have been discussed in the literature, they are unsuitable for panoramic images. Little literature [15] currently investigates the photographic composition for panoramic images.

The components deciding the types of photographic compositions consist of the line direction, the intersection points, and the relative position of objects inside the photo. The features of skin color, intensity distribution, and Canny texture are employed by Tan et al. [16] to describe and determine the image structure. Also, the salient region in the image is used to identify the photographic composition [17,18]. Based on salient points, salient line segments, and diagonal lines, Mitarai et al. [19] proposed an interactive system of shooting assistance to identify the photographic composition. However, those methods are designed to assist the photographer by considering only a few types of photographic compositions.

Recently, Chang et al. [20] proposed a photography recomposition method that automatically transfers the composition type of a reference image to an input image. An in-depth study about taking a good picture was proposed in [21], especially for photographic composition. Chang and Chen [22] proposed a stochastic search algorithm to create an exemplary view configuration within a panoramic scene. The reference images with similar compositions are selected from masterpiece photographs. Then, those configurations are used to help make professional-like photo compositions. However, those methods only consider the photographic composition based on the case of traditional images. In addition, some automatic identification methods for modifying the photo composition are proposed [20,22]. At first, salient regions such as subjects are extracted from the image and then their locations are changed to match the predetermined types of photo compositions, for example, the center composition and rule of thirds.

## 3. Proposed Scheme

### 3.1. Photographic Composition of the Panoramic Image

Professional photographers generally consider composition to be one of the critical elements for a good picture. Because the size difference between the traditional and panoramic images is large, the photographic composition for traditional images may be unsuitable for panoramic images. For example, the technique of diagonal composition arranges main subjects along the diagonal line of the scene. However, because the area covered by a panoramic image is much larger than a traditional image, it is hard to apply the same method for traditional images to a panoramic image. Therefore, the first step of this paper aims to analyze the properties of panoramic images and summarize the types of photographic compositions suitable for panoramic images. Due to that, there is no specific database for panoramic images from public resources and the types of photographic compositions are not investigated; we needed to analyze the compositions based on the characteristics of panoramic images collected from the Internet. Also, practical features needed to be designed to describe the composition components.

**Figure 1:**
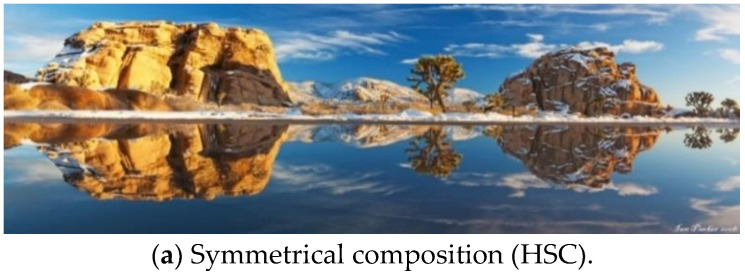

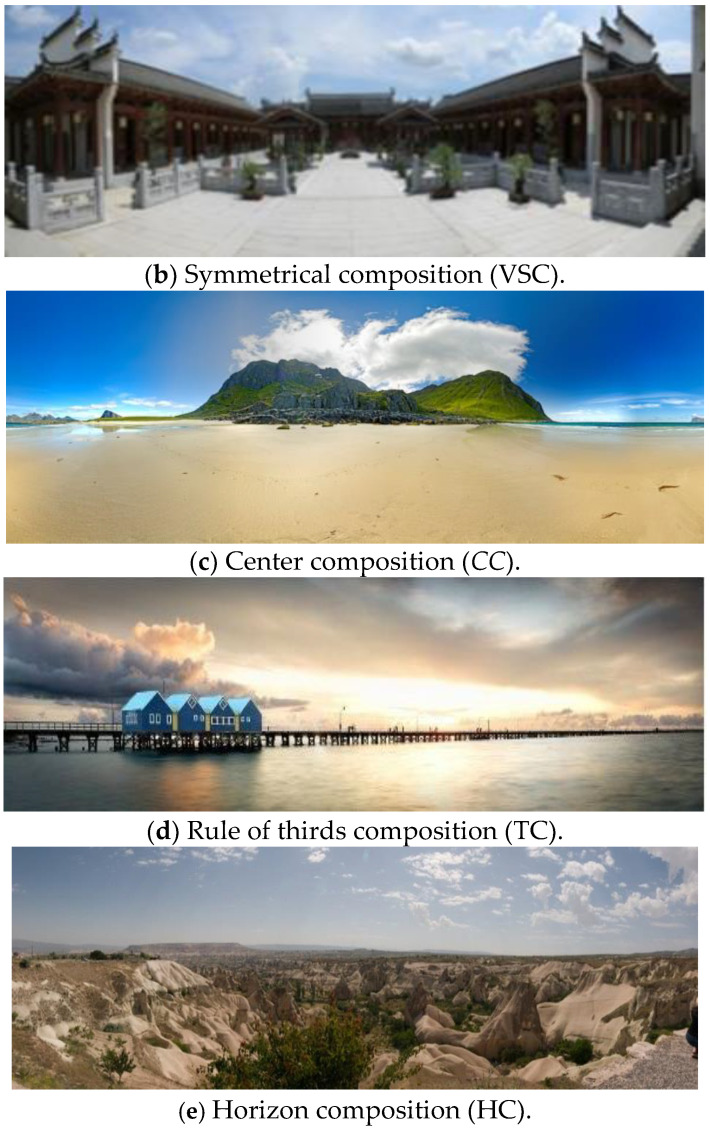
Sample panoramic images showing the five types of photo compositions.

After analyzing the collected images, five kinds of photographic compositions suitable for panoramic images were concluded. The details of each composition type are described as follows:

(1) Horizontal Symmetrical Composition (HSC)
(2) The symmetrical composition of the traditional image usually employs mirrors, water, or metal materials to generate the reflected image. There is always a horizontal or a vertical line to divide the photo into two parts, showing a symmetrical image. Therefore, this arrangement will highlight the main subject and achieve the visual balance of the photo. However, due to a wider viewing angle, the reflected surface in the panoramic image is frequently the water, i.e., lake or river, which can provide a relatively more significant reflected effect as illustrated in Figure 1a. Vertical Symmetrical Composition (VSC)
  Instead of using a horizontal line to divide the photo into two parts, another composition type called Vertical Symmetrical Composition (VSC) adopts a virtual vertical line to show a symmetrical image, which is shown in Figure 1b as an example. This virtual vertical line is usually formed by natural or artificial objects.
(3) Center Composition (CC)
  In traditional photography, the main subject is often placed in the image center, which can achieve the visual effect of emphasizing the main subject. This composition type is called the center or sun-like composition, as shown in Figure 1c. However, because the shape of the panoramic image is a long and narrow rectangle, it is hard to generate the same CC effect as a traditional photo. Therefore, apart from placing the main subject in the center of the panoramic image, the brightness and the color around the main subject should have high contrast to that of the main subject.
(4) Rule of Thirds Composition (TC)
  Rule of thirds is one of the most recognizable compositions in traditional photography. Firstly, along the horizontal or the vertical direction, the photo is divided into three equal parts by two vertical or horizontal lines. By using those four lines, the whole photo is divided into nine regions. Placing the main subject at one of those four intersection points can attract the viewer’s attention, as displayed in Figure 1d. Because the split ratio is closest to the golden ratio (1:0.618), TC is also called the golden mean composition. Given the elongated and narrow shape of panoramic images, TC proves unsuitable unless the primary subject aligns with the two vertical lines. Highlighting the main subject necessitates two conditions: (1) enhancing the brightness and color contrast around the main subject, like the CC and (2) minimizing the presence of multiple objects within the image to the greatest extent possible.
(5) Horizon Composition (HC)
  When the photographer takes a panoramic image, the camera is smoothly moved to capture the scene and the whole image is created by seamlessly stitching all sequential frames. Therefore, a horizontal line is easy to show in the image. Horizontal lines can demonstrate a stable and peaceful effect that often applies in the panoramic landscape image shown in Figure 1e. Such a photographic composition usually arranges the sky with the sea or the land to generate a horizontal line dividing the image into two regions. Hence, the apparent skyline or the horizon appears in the panoramic image as a significant characteristic of the HC. The horizontal line will be even more apparent when those two regions possess uniform color and relatively high contrast. In typical cases, the sky is placed above the horizon so that the blue color component in this region is higher than the bottom region.

### 3.2. Feature-Based Photographic Composition

As mentioned above, significant differences between various photographic compositions appear in both spatial and color domains. In this paper, a set of features is adopted to automatically identify different types of photographic compositions to determine the composition type of the panoramic image.

**Figure 2:**
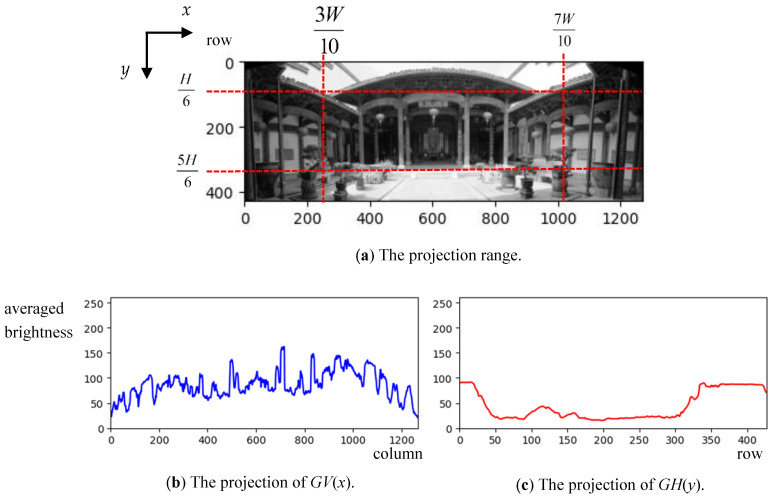
The example of the projections of *GV*(x) and *GH*(y).

**Figure 3:**
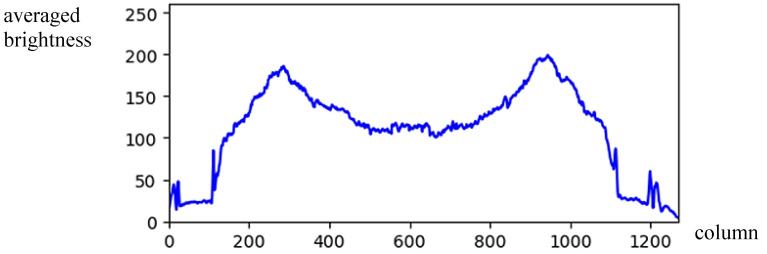
The vertical projection for the upper *H*/3 region of the image.

**Figure 4:**
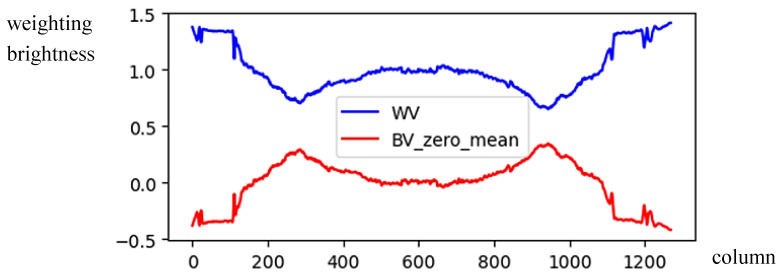
The brightness weighting function for illumination compensation in vertical projection.

**Figure 5:**
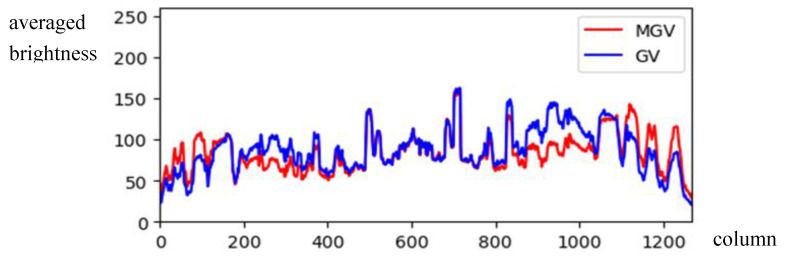
The modification of the *GV* weighted by *WV*.

**Figure 6:**
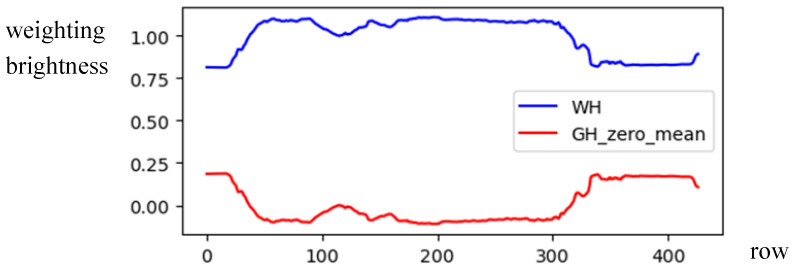
The brightness weighting function for illumination compensation in horizontal projection.

**Figure 7:**
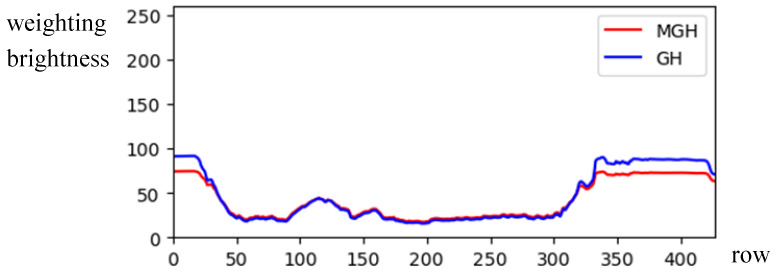
The modification of the *GH* weighted by *WH.*

**Figure 8:**
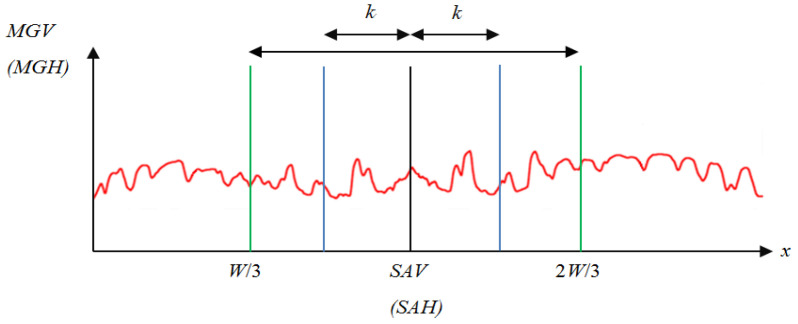
The range to search the position with the minimum value among symmetrical difference values.

**Figure 9:**
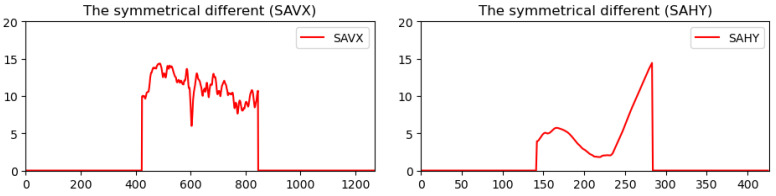
The histogram of the symmetry measurement.

**Figure 10:**
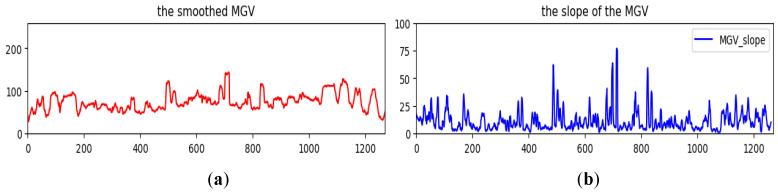
The smoothed MGV (**a**) and the original slope of the MGV (**b**).

**Figure 11:**
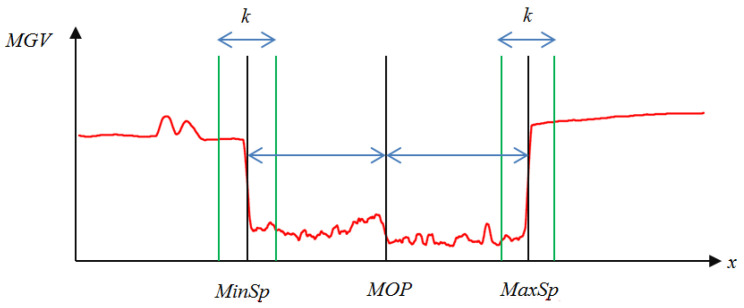
The positions of *MinSp*, *MaxSp,* and *MOP* in modified *MGV.*

**Figure 12:**
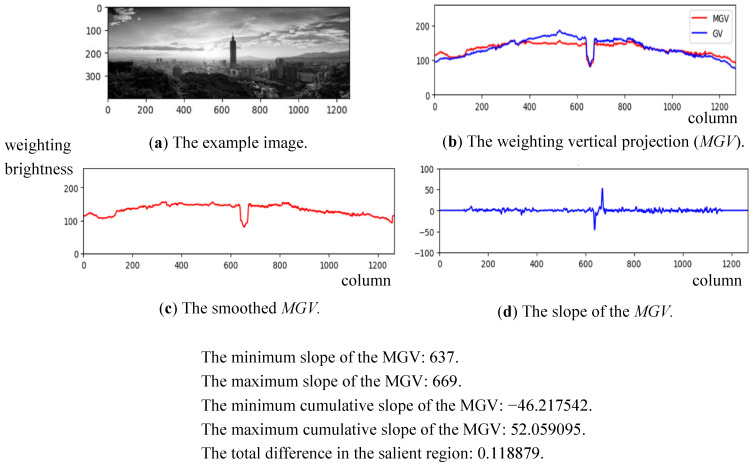
An example to show the calculation of the main subject’s position.

**Figure 13:**
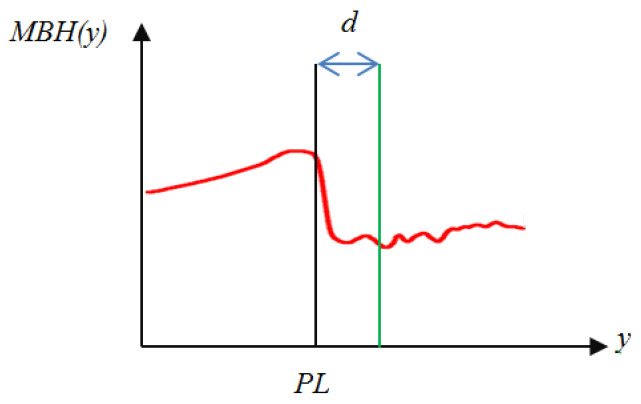
The position (*PL*) of the skyline or the horizon in the image.

**Figure 14:**
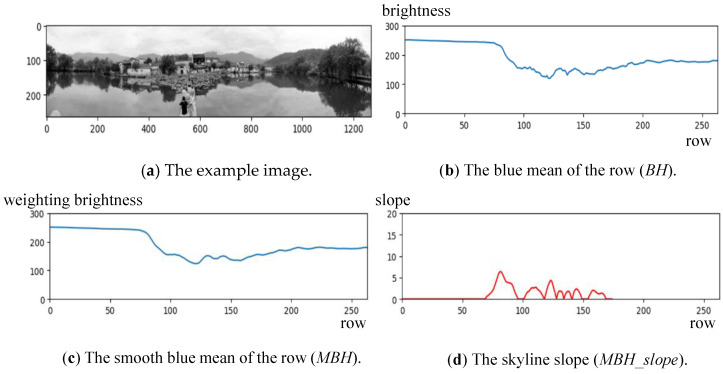
An example to show the calculation of the skyline position.

(1) *The Global Symmetry*
  The main characteristic of symmetrical composition in the panoramic image is that both the left and right (or top and bottom) areas have similar content. Hence, this feature is adopted to determine the symmetrical composition for panoramic images. The symmetry property can also be calculated by those two areas yielding the same distribution of pixel values. Let I(x,y), 1≤x≤W, 1≤y≤H be a panoramic image in which *W* and *H* are the width and height of *I*. At first, two statistical histograms are individually generated by averaging the pixel values of *I* along each column and each row. Therefore, those two histograms equal the vertical and the horizontal projections for *I*. Because messy scenes typically appear at two ends of the panoramic image that affect photo composition, we only take the projection range from *H*/6 to 5*H*/6 along the vertical direction and from 3*W*/10 to 7*W*/10 along the horizontal direction of the image. Therefore, the two histograms of *GV*(*x*) and *GH*(*y*) can be calculated by
  (1) $$GV(x)=\frac{3}{2H}\sum_{j=H/6}^{5H/6}I(x,j)$$
  and
  (2) $$GH(y)=\frac{5}{2W}\sum_{i=3W/10}^{7W/10}I(i,y)$$
  Taking Figure 1b as an example; *GV* (*x*) and *GH*(*y*) are shown in Figure 2.
  For a panoramic image, the histogram *GH* (or *GV*) will show an asymmetrical shape at the center that illustrates the similarity between the top and the bottom (or the left and the right) areas with horizontal (or vertical) symmetry. However, because the panoramic image contains expansive scenery (even with a 360-degree view), various brightness results in significant contrast appearing in different parts of the panoramic image. For example, the sunlight appearing on the left side of the image gives the right side a darker brightness, changing the histogram’s symmetry. Therefore, brightness compensation is essential for avoiding the wrong decision on symmetry in the whole panoramic image. The techniques of brightness compensation can be divided into two cases:
  (a) *The image with vertical symmetry*: Because the sun generally appears in the top area of the image, we only consider the top area’s illumination distribution, which can provide the distribution of the pixel values for the whole image. Hence, a new vertical projection for the upper *H*/3 region of the image is estimated by
    (3) $$BV(x)=\frac{3}{H}\sum_{j=1}^{H/3}I(x,j)$$
    Taking the image in Figure 1b used to calculate *GV*(*x*) and *GH*(*y*) again; the obtained *BV*(*x*) is shown in Figure 3 and the brightness average of *BV* is given by
    (4) $$MBV=\frac{1}{W}\sum_{i=1}^{W}BV(i)$$
    Figure 3 depicts the histogram of an image with vertical symmetry in which sunlight appears on the two sides. Therefore, the histogram in these locations has higher pixel values than the other part. For compensating the illumination difference, the brightness weighting function WV(x) can be obtained by
    (5) $$WV(x)=1-\frac{BV(x)-MBV}{255}$$
    Using Equation (5), the illumination compensation result of Figure 3 is shown in Figure 4 and drawn by the blue line. Consequently, the histogram *GV*(*x*) can be modified by multiplying by the weight *WV*(*x*) and given by
    (6) MGV(x)=GV(x)×WV(x), x=1,…,W
    By comparing *GV*(*x*) and *MGV*(*x*) in Figure 5 for Figure 1b, the illumination *GV*(*x*) was compensated by the brightness weighting function *WV*(*x*), showing clearer vertical symmetry in *MGV*(*x*).
  (b) *The image with horizontal symmetry*: In this case, the light source, i.e., the sun, may appear anywhere in the top image region (above the horizontal level). Therefore, we only need to use the horizontal brightness distribution (i.e., *GH*) in the center part of the image to depict the illumination distribution of each column. The average value of the *GH* is computed by
    (7) $$MBH=\frac{1}{H}\sum_{i=1}^{H}GH(i)$$
    and the brightness weighting function *WH*(*y*) based on the horizontal projection *GH* can be given by
    (8) $$WH(y)=1-\frac{GH(y)-MBH}{255}$$
    As shown in Figure 6, the brightness weighting function *WH*(*y*) is drawn by a blue line. Like the case of vertical symmetry, the compensated histogram *GV* is written as
    (9) MGH(y)=GH(y)×WH(y), y=1,…,H
    By comparing *GH*(*y*) and *MGH*(*y*) in Figure 7 for Figure 1b, the illumination *GH*(*H*) was compensated by the brightness weighting function *WH*(*y*), showing clearer horizontal symmetry in *MGH*(*y*).
    For an image with asymmetrical composition, the symmetrical axis is usually arranged in the center column x=W/2 (or the center row y=H/2), and this is difficult for the photographer when he faces a panoramic scene. However, locating the accurate symmetrical axis from the panoramic image is essential for identifying the photographic composition. To address this issue, the modified brightness histogram *MGV* (or *MGH*) was adopted to compute the symmetrical difference across both sides of the histogram. The sliding window’s width was also configured to be 2*k*, as illustrated in Figure 8. To reduce the computation time, the range to search the position with the minimum value among symmetrical difference values was shrunk to a smaller area between *W*/3 and 2*W*/3 (or between *H*/3 and 2*H*/3).
    For the case of vertical (or horizontal) symmetric composition, the accurate position of the symmetric axis, denoted by *SAV* (or *SAH*), can be obtained by selecting the minimum value among symmetrical difference values defined by
    (10a) $$\begin{array}{cc} SAVX=\arg\;\underset{x}{\min} & \frac{\sum_{i=1}^{sv}\left|MGV(x-i)-MGV(x+i)\right|}{sv},\;x\in \left\{\frac{W}{5},\ldots ,\frac{4W}{5}\right\},\;sv=\frac{W}{5} \end{array}$$
    (10b) $$SAV=\left|1-\frac{2\cdot SAVX}{W}\right|$$
    (11) $$\begin{array}{cc} SAHY=\arg\;\underset{y}{\min} & \frac{\sum_{i=1}^{s}\left|MGH(y-i)-MGH(y+i)\right|}{sh},\;y\in \left\{\frac{H}{3},\ldots ,\frac{2H}{3}\right\},\;sh=\frac{H}{3} \end{array}$$
    (11a) $$SAH=\left|1-\frac{2\cdot SAVY}{H}\right|$$
    Figure 9 shows two histograms corresponding to the symmetry measurements of *SAVX* and SAHY. We can find an apparent valley near column 600 in the *SAVX* curve indicating Figure 2 has a symmetrical axis and its location. In the *SAHY* curve, we can also find a valley near row 225 that shows a symmetrical axis and its location in Figure 2.

(2) *The Local Saliency*
  For some suitable compositions, the main subject is placed in a specific photo location to emphasize the main subject. Hence, the region’s content, including the main subject, usually demonstrates significant differences in pixel value distribution from other regions. Furthermore, because the viewer’s angle of view usually follows the horizontal direction for a panoramic image, the main subject’s position is better arranged on the horizontal axis of the image. Therefore, in the vertical projection histogram, two neighboring regions in addition to the main subject will result in two abrupt brightness changes. The histogram *MGV*(*x*) is further modified to extract the salient part from the image. After a smoothing processing to reduce the ripple effect along the *MGV* curve, two positions (*MinSp* and *MaxSp*) with the minimum and the maximum slopes can be calculated by *k* = 5. The equations of *MinSp* and *MaxSp* are given by
  (12) $$MinSp=\underset{x}{\arg\min}\left(\sum_{i=0}^{k}\left(MGV\left(x+i\right)-MGV\left(x+i-1\right)\right)\right),\;x=2,\ldots ,W-k\;\mathrm{and}\;MaxSp=\underset{x}{\arg\max}\left(\sum_{i=0}^{k}\left(MGV\left(x+i\right)-MGV\left(x+i-1\right)\right)\right),\;x=2,\ldots ,W-k$$
  Taking the *MGV* in Figure 5 for further processing; the smoothed *MGV* is shown in Figure 10a, and the corresponding slope of the *MGV* is shown in Figure 10b. The midpoints of *MinSp* and *MaxSp* are denoted by *MOP*, as shown in Figure 11. Moreover, the corresponding cumulated slope related to *k* is expressed as *k* = 5. The calculations of *MinSp*, *MaxSp*, and *MOP* are defined by
  (13) $$Min\_TotalSp=\sum_{i=0}^{k}\left(MGV\left(MinSp+i\right)-MGV\left(MinSp+i-1\right)\right)\;Max\_TotalSp=\sum_{i=0}^{k}\left(MGV\left(MaxSp+i\right)-MGV\left(MaxSp+i-1\right)\right)\;MOP=\frac{MaxSp+MinSp}{2}$$
  Also, the total difference (*TDS*) between the salient region and the neighboring regions can be measured by
  (14) $$TDS=\frac{\left|Min\_TotalSp+Max\_TotalSp\right|}{Max\_TotalSp+\left|Min\_TotalSp\right|}$$
  In general, if the main object is arranged in the center (*W*/2) of the image, we call it the Central Composition (*CC*). In addition, one-third and two-thirds of composition methods locate the main object at *W*/3 and 2*W*/3 of the image. For estimating the consistency between the main subject location and the composition rule, the calculation of the location gap *LG* is given by
  (15) $$LG_{C}=\frac{\left|MOP-SL_{C}\right|}{W},\;C\in \left\{CC,TC1,TC2\right\}$$
  (15a) $$SL_{C}=\left\{\begin{array}{l} W/2,\;\;C=CC\\ W/3,\;\;C=TC1\\ 2W/3,\;C=TC2 \end{array}\right.$$
  where *SL* is the specific location based on the composition rule. For example, the *SL* is *W*/2 for the *CC* rule and *W*/3 or 2*W*/3 for the *TC*1 and *TC*2 rules, respectively. Figure 12 demonstrates an example for calculating the location of the main subject from the slope of the *MGV*. We can find a coupe pulse in the curve of the slope of the *MGV* that indicates incidents of an apparent object at the range of column 637 to column 669.

(3) *The Horizontal linearity*
  An apparent (vertical or horizontal) line appears in the panoramic image for some compositions. For example, a horizontal line with a distinct difference between two sides often appears in the image center of the horizon composition. In addition, in the top area of the horizon, i.e., sky or cloud, the color frequently shows a satiated blue or bright white. Based on this characteristic, the B-channel of the color image is adopted to extract those lines from the panoramic image. Let the intensity image from the B-channel be B(x,y), 1≤x≤W, 1≤y≤H. A histogram *BH* representing the average pixel values of each row can be produced.
  Furthermore, as shown in Figure 7, the smoothed histogram *MBH* generated from the histogram *BH* can be made to remove the noise. Using *MBH*, the skyline can be found with a high slope change in the histogram. The possible skyline can be found by
  (16) $$SL=\arg\underset{0\leq y\leq \frac{2H}{3}}{\max}\left[\frac{\left|MBH(y)-MBH(y+d)\right|}{d}\right],\;\;d=\frac{H}{50}$$
  Please note that the skyline or horizon location is limited between *H*/3 and 2*H*/3 to match the actual case. Figure 13 shows the possible skyline *PL* in the *MBH*(*y*) histogram, and Figure 14 demonstrates an example for calculating the skyline position near row 78.
  Moreover, the matching degree of the found skyline to the horizontal direction needs to be checked. An excellent panoramic image with a horizon composition will have a horizontal line to divide the image to provide a balanced vision. When the found line is not horizontal, the locations with the most significant intensity change in each column will differ. Let the most significant change in each column be *CV*(*x*), *W*/3 < x < 2*W*/3; the standard deviation of *CV* can be computed by
  (17) $$SDL=\frac{WH}{30}\sum_{i=W/3}^{2W/3}\left|\sum_{j=1}^{H/10}I(i,SL+j)-\sum_{j=1}^{H/10}I(i,SL-j)\right|$$
  A small *SDL* value represents that the found line is nearly horizontal.

(4) *The Texture Complexity*
  In a panoramic image, uniform color or texture appears in certain regions, i.e., sky, cloud, and sea, generating esthetically pleasing images. Also, the contrast between the texture and uniform regions is needed for images. For photographic composition, three types of combinations between the uniform and the texture regions are described:
  (a) *The uniform region*: To avoid a uniform region being mistaken as having good symmetry, two features are defined to measure the texture complexity of the image and assist in SC’s decision. Based on the histogram *MGV* (or *MGH*), the corresponding standard deviation *SDGV* (or *SDGH*) is computed by
    (18) $$SDGV=\frac{1}{W}\sqrt{W\sum_{x=1}^{W}{\left(MGV(x)\right)}^{2}-{\left(\sum_{x=1}^{W}MGV(x)\right)}^{2}}$$
    and
    (19) $$SDGH=\frac{1}{H}\sqrt{H\sum_{y=1}^{H}{\left(MGH(y)\right)}^{2}-{\left(\sum_{y=1}^{H}MGH(y)\right)}^{2}}$$
  (b) *The uniform regions surrounding the texture region*: Under the case of *SC*, the main subject appears in the image center, and the surrounding regions should be uniform or blurred with low texture complexity. This feature can be used to determine the composition of *SC* or *TC*. For estimating the texture complexity of the regions surrounding the main subject, the standard deviation *SDNGV* is given by
    (20) $$SDNGV=\frac{1}{W_{L}}\sqrt{W_{L}\sum_{x=1}^{W_{L}}{\left(MGV(x)\right)}^{2}-{\left(\sum_{x=1}^{W_{L}}MGV(x)\right)}^{2}}+\frac{1}{W_{R}}\sqrt{W_{R}\sum_{x=1}^{W_{R}}{\left(MGV(x)\right)}^{2}-{\left(\sum_{x=1}^{W_{R}}MGV(x)\right)}^{2}}$$
    where *W_L_* = min{*MaxSp*, *MinSp*} and *W_R_* = *W* – max{*MaxSp*, *MinSp*}.
  (c) *The uniform region and the texture region are divided by a horizontal line*: In the case of *HC*, the top area is generally a uniform region, and the bottom area is a texture region with higher contrast. Measuring the difference in texture complexity between those two regions can assist the decision of *HC*, and this feature can be calculated by
    (21) $$BC(PL)=(BC_{D}-BC_{U})\left[1+\frac{BM_{U}-BM_{D}}{256}\right]$$
    where *BM_U_*, *BM_D_*, *BC_U_*, and *BC_D_* are given by
    $$\begin{array}{l} BM_{U}=\frac{1}{PSL-d}\sum_{y=1}^{PL-d}MBH(y)\\ BM_{D}=\frac{1}{H-PSL-2d}\sum_{i=PL+d}^{H-d}MBH(y)\\ BC_{U}=\frac{1}{PSL-d}\sum_{y=1}^{PL-d}\left|MBH(y)-MBH(y+d)\right|\\ BC_{D}=\frac{1}{H-PSL-2d}\sum_{i=PL+d}^{H-d}\left|MBH(y)-MBH(y+d)\right|\\ PSL=\frac{1}{1+\underset{0\leq y\leq \frac{2H}{3}}{\max}\left[\frac{\left|MBH(y)-MBH(y+d)\right|}{d}\right]} \end{array}$$
    and *d* is a small distance value (i.e., *H*/50). By summarizing all features mentioned above, the feature vector *F* used to identify the photographic composition of a panoramic image is represented by
    (22) $$\begin{array}{c} F=(SAV,SAH,TDS,LG_{CC},LG_{TC1},LG_{TC2},PL,SDL,SDGV,SDGH,SDNGV,BC)\\ \;=(f_{1},f_{2},f_{3},f_{4},f_{5},f_{6},f_{7},f_{8},f_{9},f_{10},f_{11},f_{12}) \end{array}$$

### 3.3. Composition Identification

After feature extraction, the composition type of the panoramic image can be identified using the decision rules defined in the feature space, which are described as follows:

(1) *Identification of HSC and VSC*
  In general, the panoramic image of the *SC* (*HSC* or *VSC*) composition has a horizontal or vertical line formed at the image’s center. So, the feature *SAH* (or *SAV*) is adopted to evaluate the distance between the symmetrical axis and the image middle. Furthermore, two separate regions will demonstrate apparent texture differences in SC and the feature SDGH (or SDGV) will be used to examine the texture complexity of two regions in addition to the horizontal (or vertical) symmetrical line. If the *SAH* (or *SAV*) value is smaller than the predefined threshold value *T_SAH_* (or *T_SAV_*) and the *SDGH* (or *SDGV*) value is larger than the predefined threshold *T_SDGH_* (or *T_SDGV_*), the considered panoramic image is identified as the symmetrical composition of *HSC* or *VSC*.
(2) *Identification of CC*
  The region with the main subject to the surrounding regions should have a sharp contrast to the center composition. Also, the size of the main subject is larger than the other objects in the panoramic image. Furthermore, the main subject’s position must be close to the image center. Therefore, three rules must be satisfied by the *CC*:
  (a) *Location rule*: The feature *LG* from Equation (15) can be applied to measure the distance between the main subject and the image center (*SL*). The center position of the main subject is *MOP*, and the *SL* is set to *W*/2 in Equation (15). If the *LG* is smaller than the threshold value *T_LG_*, we can assume that the main subject is at the center of the panoramic image.
  (b) *Saliency rule*: One essential rule for *CC* is that salient texture difference exists between the region with the main subject and its surrounding regions. This rule can be measured by computing the feature *TDS* in Equation (14), and the composition belongs to *CC,* while the *TDS* value is smaller than the predefined threshold value *T_TDS_*.
  (c) *Contrast rule*: The regions surrounding the main subject will have low contrast, and the feature *SDNGV* can measure this in Equation (20). The main subject has a significant contrast with its surrounding region, while the *SDNGV* is smaller than the predefined threshold value *T_SDNGV_*.
(3) *Identification of TC*
  For a good *TC*, the main subject is located at *W*/3 or 2*W*/3 along the horizontal axis, and a salient texture difference exists between the region with the main subject and its surrounding regions. Therefore, like the identification of *CC* in 2), three features (*LG*, *TDS*, and *SDNGV*) are applied to identify the type of *TC*. The only distinction between *SC* and *TC* is that the *SL* is set to *W*/3 or 2*W*/3 in Equation (15).
(4) *Identification of HC*
  A panoramic image belonging to the type of HC should satisfy the following three rules:
  (a) A manifest horizontal line exists between the sky and land or sea regions. The feature *SL* in Equation (16) is adopted as the possible position of the skyline.
  (b) The partition line (or skyline) must be horizontal, and this characteristic is fulfilled, while the feature value *SDL* computed from Equation (17) is larger than a threshold value *T_SDL_*.
  (c) The top region above the horizon is frequently the sky or the cloud with uniform and bright intensity. The texture content at the bottom side under the horizon is significantly different from the top region, and the characters can be evaluated using the feature *BC* defined in Equation (21).

**Figure 15:**
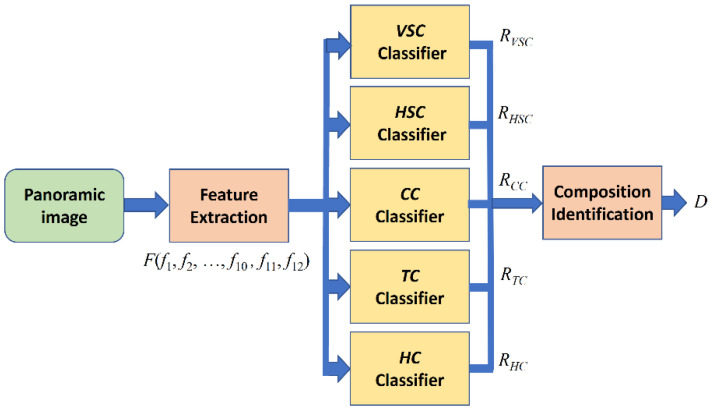
The schematic representation of our proposed framework.

The relationship among the ten major features and five composition types is summarized in Table 1. Note that the feature *LG* consists of the three features of *LG_CC_*, *LG_TC1_*, and *LG_TC2_*. In this paper, after feature extraction from each panoramic image, twelve feature values are combined into a feature vector *F* defined in Equation (22). The proposed framework for the composition identification is organized by five classifiers corresponding to each composition, and the schematic representation of our proposed framework is shown in Figure 15.

The output of each classifier is a response vector represented by

(23) $$R_{j}=(r_{j1},r_{j2},r_{j3},r_{j4},r_{j5},r_{j6},r_{j7},r_{j8},r_{j9},r_{j10})$$

where *j* is one element from the set of photo compositions *C* = {*VSC*, *HSC*, *CC*, *TC*, *HC*},

(24) $$r_{ji}=\left\{\begin{array}{c} 1,\;f_{i}\in T_{ji}\;\mathrm{or}\;T_{ji}=Null\\ 0,\;f_{i}\notin T_{ji} \end{array}\right.i=1,2,\ldots ,10$$

and *T_ji_* is the range determined by the *i*th threshold value of the *j*th composition type. All used threshold values are represented as a threshold vector:

(25) $$\begin{array}{c} T_{j}=(T_{j,SAV},T_{j,SAH},T_{j,TDS},T_{j,LG},T_{j,PL},T_{j,SDL},T_{j,SDGV},T_{j,SDGH},T_{j,SDNGV},T_{j,BC})\\ \;=(T_{j1},T_{j2},T_{j3},T_{j4},T_{j5},T_{j6},T_{j7},T_{j8},T_{j9},T_{j10}) \end{array}$$

For an input panoramic image, the composition identification is achieved by using those five response vectors from Equation (23) as the input and generating the vector of composition types as the output given by

(26) $$D=(c_{VSC},c_{HSC},c_{CC},c_{TC},c_{HC})$$

where *c_j_* is assigned by *Rule_j_*, as explained in Section 3.4.

### 3.4. Composition Identification Using Fuzzy Rules

The composition type included in the panoramic image can be identified using the features defined in Section 3.2 and the decision rules described in Section 3.3. However, multiple types may exist in an image simultaneously, which also happens when a photography expert evaluates a panoramic image. Novel approaches are further proposed in this section to evaluate different composition types using fuzzy rules for solving the problem that the proposed framework produces only one type of identification result. Hence, multiple types of compositions are allowed in a panoramic image. Twelve feature values are extracted for each panoramic image and combined into a feature vector *F* in Equation (22). After those features are converted into fuzzy features, they are further calculated using the proposed set of fuzzy rules. The final output is the possible types of compositions by referring to the attributes of fuzzy classes.

**Figure 16:**
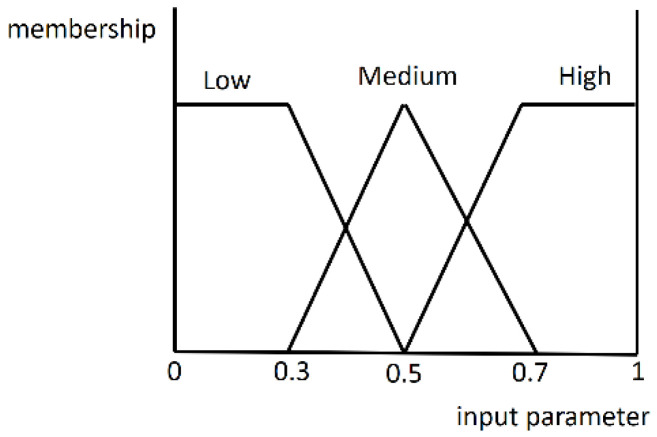
The membership function to convert each feature value.

Based on the Mamdani-style inference system the twelve feature values obtained are further individually converted into the [0, 1] interval. The fuzzifier function is defined by

(27) $$F_{L}=\frac{1}{1+e^{-a(X_{i}-X_{T})}}\;\;(X_{T}=0.5,\;\alpha =5)$$

and

(27a) $$F_{S}=1-\frac{1}{1+e^{-a(X_{i}-X_{T})}}\;\;X_{T}=30,\;\alpha =1/5)$$

where *X_i_* is the *X* feature value in the *i*-th image and *X_T_* is the predefined parameter. The curve slope is represented by a. Furthermore, the function *F_L_* is used to fuzzify the feature values of *SAV*, *SAH*, *TDS*, *LG_CC*, *LG_TC1*, *LG_TC2*, and *PL*, and the function *F_S_* is adopted to fuzzify the feature values of *SDL*, *SDGV*, *SDGH*, *SDNGV*, and *BC*. The membership function used is shown in Figure 16.

The rules corresponding to each photographic composition, which are used to create the fuzzy logic rules for composition identification, are described as follows:

(1) Identification of TC
  For the VSC composition, because the color symmetry appears in the middle of two image regions in addition to the vertical line, the image should have global symmetry with a low degree (*SAV*(*L*)). Also, to avoid the wrong *VSC* decision caused by uniform color content, the image should have the global and horizontal texture complexity above the middle degree (*SDGV*(*M*) and *SDGV*(*H*)).
  ***Rule****_VSC_* = ***Min***{*SAV*(*L*), ***Max***{*SDGV*(*M*), *SDGV*(*H*)}} (28)
(2) Identification *of HSC*
  For the *HSC* composition, because the color symmetry appears in the middle of two image regions in addition to the horizontal line, the image should have global symmetry with a low degree (*SAH*(*L*)). Also, to avoid the wrong *HSC* decision caused by uniform color content, the image should have the global and horizontal texture complexity above the middle degree (*SDGH*(*M*) and *SDGH*(*H*)).
  ***Rule****_HSC_* = ***Min***{*SAH*(*L*), ***Max***{*SDGH*(*M*), *SDGH*(*H*)}} (29)
(3) Identification *of CC*
  For the *CC* composition, because the main subject appears in the image center (*LG*(*L*)) with prominent contrast to the surrounding regions, the image should have local saliency with a low degree (*TDS*(*L*)). Also, to emphasize the main subject, the texture complexity of the other objects must be shallow (*SDNGV*(*L*)).
  ***Rule****_CC_* = ***Min***{*LG*(*L*), *TDS*(*L*), *SDNGV*(*L*)} (30)
(4) Identification *of TC*
  For the *TC* composition, the main subject appears at *W*/3 or 2*W*/3 along the horizontal axis (*LG*(*L*)), and a salient texture difference exists between the region with the main subject and its surrounding regions. Therefore, the image should have local saliency with a low degree (*TDS*(*L*)) and the texture complexity of the other objects must be very low (*SDNGV*(*L*)).
  ***Rule****_TC_* = ***Min***{*LG*(*L*), *TDS*(*L*), *SDNGV*(*L*)} (31)
(5) *Identification of HC*
  For the *HC* composition, because two regions with significant color differences appear above and under the skyline in the image, the image should have skyline linearity above the middle degree (*PL*(*M*) and *PL*(*H*)). Also, skyline levelness should be checked (*SDL*(*H*)). Furthermore, the texture complexity of the upper region above the skyline is lower than that of the bottom region (*BC*(*H*)).
  ***Rule****_HC_* = ***Min***{***Max***{*PL*(*M*), *PL*(*H*)}, *SDL*(*H*), *BC*(*H*)} (32)
  Based on those five rules defined from Equation (28) to Equation (32), each panoramic image’s degree of composition membership can be calculated and used as the output value to decide the photographic composition. Each degree value’s range will be located at [0, 1].

## 4. Experimental Results

This section evaluates a test image database including 168 panoramic images for the composition types by the proposed approaches. For verifying the accuracy of the proposed system, this database was evaluated by photography experts, and the identification results are recorded as the benchmark before the system identification.

### 4.1. Experiment 1: Composition Identification Using Feature Vectors

**Figure 17:**
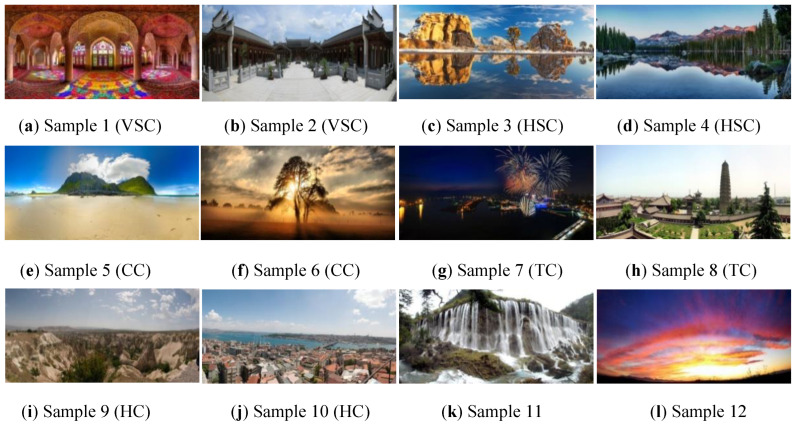
The test set of 12 panoramic images.

In Experiment 1, two sample images were selected from each class in the image database. The total number is 12, and all sample images are shown in Figure 17 in which Samples 1 and 2 are vertical *SC*; Samples 3 and 4 are horizontal *SC*; Samples 5 and 6 are *CC*; Samples 7 and 8 are *TC*; Samples 9 and 10 are *HC*; and Samples 11 and 12 are incompatible composition.

For the feature vector in Equation (22), the optimized threshold values in Equation (25) are described as:

*T_TC_* = (17, 19, 72, 0.14, 35, 0.25, 20, 20, 0.22, 1.4);

*T_CC_* = (17, 19, 72, 0.08, 35, 0.25, 20, 20, 1.3, 1.4);

*T_SC_* = *T_HC_* = (17, 19, , , 35, 0.25, 20, 20, , 1.4).

The feature values extracted from the 12 sample images were evaluated and are shown in Table 2. Table 3 lists the comparison results of our identification system to the human experts. The results demonstrate that our identification system can perform similarly to human experts. Especially when multiple composition types exist for Samples 5 and 6, as decided by the experts, the proposed system can also provide the correct results.

To evaluate the effectiveness and efficiency of the proposed method, an extensive experiment was conducted on the test database with 168 panoramic images. The metrics of Sensitivity and Precision were calculated by True Positive (*TP*), False Positive (*FP*), and False Negative (*FN*) and given by

(33) $$Sensitivity=\frac{TP}{TP+FN}$$

(34) $$Precision=\frac{TP}{TP+FP}$$

Table 4 summarizes the evaluation results of the 168 panoramic images using the optimized threshold values. Each image was labeled a proper composition by photographic experts, and this result was used as the ground truth. The experimental results show that the achieved performance for the sensitivity and accuracy of the proposed method is more than 90% and at least 87%.

### 4.2. Experiment 2: Composition Identification Using Fuzzy Rules

For evaluating the effectiveness and efficiency of the proposed method using fuzzy rules, the test database with 168 panoramic images was also used. Half of the database was adopted as the training set and calculated the curve slope *a* and the predefined parameter *X_T_* in Equation (27). Furthermore, the set of 12 panoramic images in Figure 17 was used again as the test set. The characteristic fuzzy numbers computed from the test set with 12 images are listed in Table 5.

Moreover, based on the designed fuzzy rules from Equation (28) to Equation (32) for each composition, the membership grades calculated for the 12 test images are listed in Table 6. Note that the range of each membership grade is between 0 and 1. As shown in Table 3 and Table 6, after comparing the identification results from the human experts to that of the proposed system, we can find that the membership grades obtained from the proposed system can achieve high values for those test images with the same composition types as the human experts. Also, for the test images with two composition types, such as Sample 5 and Sample 6, high membership grades in two individual types can be accomplished simultaneously. Furthermore, for the composition type *HC* neglected by the human experts in Sample 8, the proposed system can provide a high membership grade to remind human experts.

Due to the page limitation of the paper, although all the membership grades for each composition type calculated for the test database with 168 panoramic images cannot be listed, the experimental results demonstrate that appropriate values were achieved and they coincide with the decision of the human experts.

## 5. Conclusions

In this paper, based on color, structure, and texture features extracted from the images, a novel approach of composition identification using fuzzy rules for panoramic images is proposed. The characteristics related to photographic composition are summarized from the analysis of the database of panoramic images. At first, the five most common types of photographic compositions are concluded. Furthermore, a feature vector with twelve feature values extracted from the image’s color, structure, and texture is designed. Based on the feature vector calculated from each panoramic image, correct composition types can be decided on and evaluated by human experts.

Because multiple composition types may exist simultaneously in an image, the proposed approaches were modified using fuzzy rules. After those feature values were converted into fuzzy feature values, they were further calculated using the set of fuzzy rules. The final output is the possible types of compositions by referring to the attributes of fuzzy classes. Hence, the modified approaches can also decide on each composition type represented by different membership grades corresponding to the identification results of human experts.

The experimental results show that both methods (with/without fuzzy rules) have demonstrated promising performance in composition identification for the test database with 168 panoramic images. In the future, based on the approaches proposed in this paper, an esthetic judgment system can be designed to evaluate the artistic value of the photographic composition for panoramic images. Also, this system can be extended to guide the photographer in shooting a panoramic image with good composition.

## Figures and Tables

**Table 1:** Relationship among the ten features and the composition types.

Photographic Composition		*HSC*	*VSC*	*CC*	*TC*	*HC*
Global Symmetry	*SAV*		●			
	*SAH*	●				
Local Saliency	*TDS*			●	●	
	*LG*			●	●	
Horizontal Linearity	*PL*					●
	*SDL*					●
Texture Complexity	*SDGV*		●			
	*SDGH*	●				
	*SDNGV*			●	●	
	*BC*					●

**Table 2:** Feature values extracted from the 12 test images.

Sample	*SAV*	*SAH*	*TDS*	*LG*	*PL*	*SDL*	*SDGH*	*SDGV*	*SDNGV*	*BC*
1	5.824	14.743	26	0.077	11.651	0.197	17.457	37.27	1.509	0.328
2	14.026	27.59	27	0.029	19.123	0.212	47.519	81.453	8.423	1.009
3	11.035	11.276	20	0.053	26.965	0.176	39.402	18.556	1.008	−0.446
4	24.113	5.133	61	0.113	9.283	0.211	30.789	46.687	0.691	0.874
5	6.98	6.916	83	0.021	32.752	0.202	17.437	63.478	0.196	1.01
6	8.467	9.739	106	0.044	13.751	0.172	6.363	63.088	1.026	−0.419
7	25.019	8.2	87	0.025	8.62	0.212	14.991	31.619	0.199	−0.685
8	19.296	37.490	89	0.001	34.65	0.441	29.091	40.356	0.005	2.744
9	3.745	42.361	9	0.025	47.673	0.150	12.47	2.275	2.961	1.474
10	8.633	19.439	4	0.036	45.4	0.176	12.016	8.2696	2.575	2.281
11	19.565	15.061	102	0.001	13.743	0.325	6.082	45.995	0.997	0.173
12	3.358	28.628	8	0.041	10.213	0.221	20.787	4.406	0.747	0.794

**Table 3:** The comparison of the proposed system to the human experts. (Experts/System).

	Composition	*VSC*	*HSC*	*CC*	*TC*	*HC*
Sample						
1		1/1	0/0	0/0	0/0	0/0
2		1/1	0/0	0/0	0/0	0/0
3		0/0	1/1	0/0	0/0	0/0
4		0/0	1/1	0/0	0/0	0/0
5		1/1	0/0	1/1	0/0	0/0
6		1/1	0/0	1/1	0/0	0/0
7		0/0	0/0	0/0	1/1	0/0
8		0/0	0/0	0/0	1/1	0/0
9		0/0	0/0	0/0	0/0	1/1
10		0/0	0/0	0/0	0/0	1/1
11		0/0	0/0	0/0	0/0	0/0
12		0/0	0/0	0/0	0/0	0/0

**Table 4:** Performance analysis for the test database.

Composition	*TP*	*FP*	*FN*	Sensitivity	Precision
Horizontal *SC*	17	2	1	0.94	0.89
Vertical *SC*	37	5	4	0.90	0.88
*CC*	10	1	0	1.00	0.91
*TC*	9	0	1	0.90	1.00
*HC*	48	7	5	0.91	0.87

**Table 5:** Fuzzy numbers calculated for the 12 test images.

Sample	*SAV*	*SAH*	*TDS*	*LG*	*PL*	*SDL*	*SDGH*	*SDGV*	*SDNGV*	*BC*
1	0.982	0.235	0.695	0.595	0.018	0.701	0.007	0.649	0.604	0.024
2	0.843	0.422	0.447	0.768	0.063	0.692	0.969	0.987	0.000	0.308
3	0.002	0.840	0.529	0.775	0.203	0.713	0.981	0.274	0.424	0.001
4	0.238	0.917	0.570	0.720	0.012	0.693	0.832	0.804	0.539	0.201
5	0.975	0.442	0.751	0.791	0.405	0.698	0.007	0.944	0.707	0.309
6	0.962	0.863	0.838	0.717	0.026	0.716	0.001	0.942	0.728	0.001
7	0.195	0.404	0.768	0.814	0.011	0.692	0.003	0.534	0.706	0.001
8	0.549	0.134	0.859	0.843	0.485	0.675	0.162	0.653	0.761	0.998
9	0.989	0.108	0.401	0.779	0.895	0.723	0.001	0.087	0.612	0.761
10	0.961	0.662	0.373	0.746	0.854	0.713	0.001	0.136	0.741	0.989
11	0.530	0.228	0.825	0.841	0.026	0.620	0.001	0.794	0.428	0.012
12	0.990	0.391	0.340	0.728	0.014	0.686	0.019	0.102	0.587	0.152

**Table 6:** Composition membership grades of the 12 test images.

Sample	*VSC*	*HSC*	*CC*	*TC*	*HC*
1	0.746	0	0.478	0.205	0
2	1	0	0	0	0
3	0	1	0	0	0
4	0	1	0	0.198	0
5	1	0	1	0.163	0
6	1	0	1	0.185	0
7	0	0	0	1	0
8	0.247	0	0.261	1	0.875
9	0	0	0	0	1
10	0	0	0	0	1
11	0.153	0	0	0	0
12	0	0	0	0	0

## Data Availability

All data sources are contained within the article.
